# Impact of Tuberculosis on the Quality of Life

**DOI:** 10.4103/0970-0218.39249

**Published:** 2008-01

**Authors:** Meera Dhuria, Nandini Sharma, GK Ingle

**Affiliations:** Department of Community Medicine, Maulana Azad Medical College, Bahadurshah Zafar Marg, New Delhi - 110 092, India

## Introduction

Tuberculosis (TB) control has been accorded a high priority within the health sector as it is a major public health problem. The Revised National Tuberculosis Control Programme (RNTCP) with Directly Observed Treatment Short Course (DOTS) as the strategy was introduced in 1997. The programme uses sputum negativity and weight gain as prognostic indicators but does not consider any other dimension of health. It has been seen that apart from physical symptoms, TB patients face various problems that are social and economic in nature. Therefore, for a comprehensive assessment of patients' health status, it is essential to consider the overall impact of TB on health and patients' perception of well being, besides routine clinical, radiological and bacteriological assessments.(1)

This can be performed by measuring the Quality of Life (QoL) that has several dimensions. The effect of disease on each dimension can be assessed using instruments, which are either generic or specific. The impact of various chronic diseases like hypertension, leprosy and asthma and depression has been studied using these instruments. In the present study, WHO QoL (BREF)(2) which has the four domains was used to assess the impact of TB on the QoL. It is required to incorporate the measurement of QoL of TB patients to have an in-depth understanding of the effect of disease on various dimensions of health. This would enable the health care professionals and the system to devise relevant interventions to improve the quality of the programme.

## Materials and Methods

Patients with tuberculosis of category I, II and III aged 20-65 years, who registered at the DOT centres between March 2004 and May 2004 were included in the study. Patients who had known respiratory co-morbidity other than TB or any known and diagnosed chronic illness, which may affect QoL, were excluded from the study. Controls were selected from general population of areas catered to by the DOT centres after matching for age, gender and socio-economic status. A total of 90 cases who met the inclusion criteria were interviewed at the study centre itself within first three days of registration using a pre-designed, pre-tested questionnaire regarding socio-demographic data, perception about his QoL using WHO QoL BREF (Hindi version) which is a 26-item scale designed by WHO. It has four domains viz: physical health, psychological health, social relationships, environment. Data were entered in MS excel and analyzed using SPSS software for windows. For comparison between mean scores of groups, independent *t*-test was used. The mean scores for different domains were used in the analysis. The overall QoL was assessed using specific questions and the mean scores for it were not the average of the mean scores of the domains.

## Results

Out of total 90 patients with TB who were included in the study, 51 (56.7%) were males and 39 (43.3%) were females. Majority (70%) of the patients belonged to 20-39 years age group (mean age 31.19 ± 10.25 years) were Hindus and married. Almost half (48%) of the patients were illiterate. The mean per capita income per month of the study subjects was Rs. 620 ± 430. The socio-demographic profile of the controls was comparable to that of the cases with no significant difference (*P* > 0.05). Almost half (48.9%) of the total 90 patients who were included in the study belonged to Cat I, one-third (30%) to Cat III and about one-fifth were of Cat II.

The TB patients had significantly lower mean scores than the controls for overall QoL and its domains. The most affected domains were physical and psychological. The patients scored highest in the Social domain (14.35 ± 2.50). The mean difference in scores for the cases and the controls was highly significant for all the domains and the overall QoL; physical (mean diff. 2.79), psychological (mean diff. 3.26), Social (mean diff. 1.33), environmental (mean diff. 2.60) and overall QoL (mean diff. 3.23) as shown by the *t*-test for independent samples [Table 1]. It can be inferred that the QoL of TB patients was lower than the controls and all domains of QoL are affected in TB.

**Table 1:** Mean quality of life scores by domains of quality of life

Domain	Physical Mean (SD)	Psychological Mean (SD)	Social Mean (SD)	Environmental (SD)	Overall QOL Mean (SD)
Cases *N* = 90	9.32(1.73)	9.82(1.98)	14.35 (2.50)	11.43(1.61)	10.98(1.40)
Controls *N* = 90	12.11 (0.91)	13.08(1.37)	15.68(1.74)	14.03(1.23)	14.21 (1.00)
Mean difference	2.79	3.26	1.33	2.60	3.23
*t*-value	15.11	12.84	4.12	12.13	15.45
*P*-value	0.000	0.000	0.000	0.000	0.000

Females had a lower mean score (11.67 ± 1.26) than the males (11.85 ± 1.66) for overall QoL and the social domain (male vs. female: 14.59 ± 2.35 vs. 14.38 ± 2.49). For the physical and environmental domain, females fared significantly better than the males (Male vs. female: Physical 9.00 ± 1.46 vs. 9.74 ± 2.04, environmental 11.26 ± 1.52 vs. 11.72 ± 1.77).

## Discussion

The QoL assessed for the cases when compared with that of the control group helped in the evaluation of the impact of TB on QoL of patients. QoL is defined as a person's perception of his or her physical and mental health and covers broad domains including physical, psychological, economic, spiritual and social well being.(3) Health care to be comprehensive in true sense must include not only the indicators of changes in frequency and severity of disease but also an estimation of well being.

The TB patients had significantly lower mean scores than the controls for overall QoL and its domains. The worst affected were physical domain followed by the psychological domain. A study(4) carried out in USA had also shown that patients had problems with physical functioning that involves one's ability to carry on normal physical activities. In China, a study conducted on TB patients using SF-36 questionnaire also showed that health-related QoL declines in patients having TB with physical scales the most affected.(5)

Tuberculosis is a disease with social implications due to the stigma attached to it which is evident from the lower scores of cases in psychological and social domains. This is in coherence with the other studies(4,5) which point out that TB affects all the predicted domains of QoL. i.e. psychological, general health perception and social role functioning.

The environmental domain relates to the sense of safety, security, home environment, transport and financial security which was negatively affected in TB patients.

Khan *et al*., had found in a study conducted in Pakistan on socio-cultural constraints in treatment that while both male and female TB patients face social and economic problems, female patients are more affected.(6) This is in coherence with the results of our study.
